# Eosinophil ETosis and Cancer: Ultrastructural Evidence and Oncological Implications

**DOI:** 10.3390/cancers17193250

**Published:** 2025-10-07

**Authors:** Rosario Caruso, Valerio Caruso, Luciana Rigoli

**Affiliations:** 1 Department of Human Pathology in Adult and Developmental Age “G. Barresi”, University of Messina, 98123 Messina, Italy; 2 School of Advanced Studies, University of Camerino, 62032 Camerino, Italy

**Keywords:** gastric carcinoma, eosinophils, ETosis, tumor-associated tissue eosinophilia, ultrastructure

## Abstract

Eosinophils are innate immune cells that infiltrate tissues in response to cell proliferation and necrosis, which occurs during normal injury repair, parasitic infections, allergies, and cancer. Their involvement in cancer is controversial particularly with regard to tumor-associated tissue eosinophilia (TATE) and a recently defined mechanism of extracellular trap cell death (ETosis), a particular type of eosinophil cell death that is distinct from both apoptosis and necrosis. This narrative review synthesizes the literature regarding the prognostic significance of TATE, focusing on eosinophil ETosis and the important role of transmission electron microscopy (TEM) in its detection and morphological characterization. The prognostic role of TATE is contradictory: in certain tumors, it is a favorable prognostic marker, while in others, it is unfavorable. However, recent research reveals that TATE is associated with a better prognosis in non-viral neoplasms, but it may correlate with a poor prognosis in virus-related neoplasms, such as human T-lymphotropic virus type 1 (HTLV-1)-associated lymphomas and HPV-positive carcinomas. Our ultrastructural investigations revealed distinct phases of eosinophil ETosis in gastric cancer, which were defined by chromatin decondensation, plasma membrane disruption, granule discharge, and development of extracellular traps. We observed synapse-like interactions between eosinophils, exhibiting ETosis or compound exocytosis, and tumor cells, which showed various degrees of cellular damage, ultimately leading to colloid-osmotic tumor cell death. TEM provides important insights into eosinophil-mediated cytotoxicity, requiring further investigation as potential immune effector mechanisms in non-viral tumors. TATE evaluation, together with the viral status of the neoplasia, may be useful to confirm its prognostic significance and consequently its therapeutic implication in specific cancers.

## Simple Summary

Eosinophils are multifunctional innate immune cells involved in inflammatory responses, tissue remodeling, and antitumor immunity. The infiltration of eosinophils in tumors, known as tumor-associated tissue eosinophilia (TATE), is frequently linked to improved prognosis in non-viral malignancies. In cancers that are caused by viruses, such as human papilloma virus (HPV)-related carcinomas and human T-lymphotropic virus type 1 (HTLV-1)-related lymphomas, TATE may be linked to adverse outcomes. However, the results reveal heterogeneity among tumor types, and no meta-analytic data is now available. This review examines eosinophil extracellular trap cell death (ETosis), a recently identified cytolytic mechanism through which eosinophils release DNA traps and cytotoxic granules. This study uses transmission electron microscopy (TEM) to provide new ultrastructural details of ETosis in human gastric cancer, emphasizing potential mechanisms of eosinophil-mediated tumor cytotoxicity. The data suggest that eosinophils may fight cancer by directly interacting with tumor cells. Future studies should include histological evaluation of TATE and standardized viral testing in virus-driven neoplasms to consolidate the prognostic value of TATE in specific cancers.

## 1. Introduction

Eosinophils are granulocytic innate immune cells that come from the bone marrow. In healthy individuals, they constitute about 3–5% of all circulating leukocytes [1]. Eosinophils, classically linked to allergy responses and antiparasitic defense, are now considered multifunctional cells that may play a role in tissue remodeling and anticancer immunity [2]. From an ultrastructural viewpoint, these innate immune cells display bilobed nuclei with dense peripheral heterochromatin and centrally located euchromatin [3]. Each eosinophil possesses approximately 200 specific granules [4] that contain four distinct cationic proteins, such as major basic protein, eosinophil cationic protein, eosinophil peroxidase, and eosinophil-derived neurotoxin, as well as various cytokines, chemokines, and growth factors [3,5,6,7]. Their effector activities include the production of reactive oxygen species through respiratory burst, the synthesis of lipid mediators, and the regulated release of both pre-formed and newly synthesized mediators through degranulation [3].

Transmission electron microscopy (TEM) is still an important tool for studying eosinophils, despite its inherent limitation of examining relatively small tissue areas. TEM provides detailed information about important aspects of eosinophil biology, such as the structure of granules, the various mechanisms of degranulation, and intercellular interactions [3,8,9,10].

**Figure 1:**
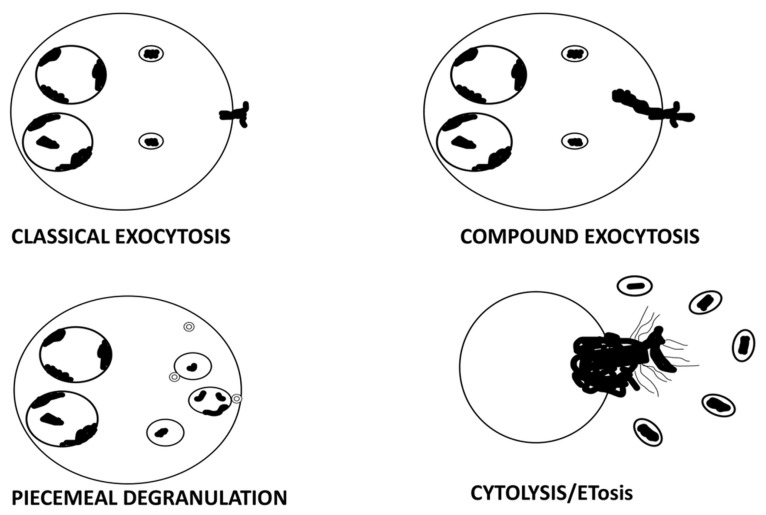
Schematic representation of the main patterns of eosinophil degranulation. Eosinophils can release their granule contents through classical exocytosis, compound exocytosis, piecemeal degranulation, or cytolysis/ETosis.

Degranulation may proceed through several pathways: classical exocytosis, compound exocytosis, piecemeal degranulation, and cytolytic release of intact granules [3]. Recent research has identified extracellular trap cell death (ETosis) as an advanced process of eosinophil cytolysis [11]. More specifically, it is a type of programmed cell death that entails the discharge of cellular contents, such as specific granules and nuclear chromatin, in the form of extracellular traps (Figure 1) [12,13,14]. Most of the research on ETosis has been done on eosinophil-related disorders in vivo and in vitro [15,16]. However, very little is known about its occurrence and functional importance in cancer.

Lowe et al. [17] created the term “tumor-associated tissue eosinophilia” (TATE) to refer to an elevated eosinophil density in the peritumoral and intratumoral inflammatory infiltrate. The role of TATE remains controversial, since it has been characterized as either prognostically favorable or unfavorable [18,19,20]. Furthermore, little is known about the precise processes by which eosinophils inhibit the growth of human tumors and the direct evidence of their anticancer impact. This narrative review synthesizes key studies regarding the prognostic value of TATE and analyzes the reasons for their controversial significance in the literature. In addition, we conducted a retrospective evaluation of our ultrastructural institutional experience with human gastric cancers characterized by TATE [21,22,23,24,25,26,27,28,29,30]. This review details the mutual membrane contact between eosinophils and tumor cells and the alterations that occurred during the interaction. The objective of this study is to clarify the potential antitumoral function of activated eosinophils and to suggest future research directions in the field of oncology.

## 2. Methodological Considerations

This review is narrative and is not organized according to the formal structure of a systematic review. However, in order to guarantee transparency, we have delineated the primary criteria that were employed for the literature search and selection. An extensive search was performed in the PubMed, Google Scholar and Scopus databases from January 1990 to April 2025, with the following keywords and their combinations: eosinophil, ETosis, extracellular traps, gastric cancer, tumor microenvironment, and ultrastructure. Only peer-reviewed articles in English were considered. Studies were included if they focused on eosinophil ETosis in cancer, specifically with ultrastructural or morphological observations. To furnish a more comprehensive biological and clinical context, investigations into TATE, tumor-associated blood eosinophilia (TABE), eosinophil-mediated cytotoxicity, and eosinophil participation in virus-associated neoplasms were also evaluated. Articles not related to eosinophils in oncology were excluded. In addition to published literature, we included ultrastructural observations from our previous research. Specifically, this review summarizes salient ultrastructural features already reported in two case reports: one that describes eosinophil exocytosis in conjunction with injury to individual gastric carcinoma cells [28], and another that details eosinophil ETosis in relation to single tumor cell damage [29]. We also examined a case series that compared the ultrastructural characteristics of non-ETotic cytolysis in three gastric cancer examples with ETotic cytolysis within the same case, as previously documented [30]. The main clinicopathologic findings of these cases are detailed in the three publications [28,29,30]. With respect to figure originality, Figures 3–6 are analogous, though not identical, to images previously published [30], while Figure 7 is analogous, though not identical, to images published in [28]. In contrast, Figures 8–10 are completely novel, derived from additional ultrastructural investigations performed on the case of gastric cancer characterized by eosinophil ETosis [29,30]. Statistical correlations between eosinophil counts, ETosis frequency, and tumor features were not feasible due to the very limited number of ultrastructural cases, with eosinophil ETosis documented in only one case. Larger cohorts will be needed to address this point. The gastric cancer cases analyzed were obtained from archival diagnostic material collected from 1998 to 2005, before the implementation of molecular testing for viral pathogens at our institution. Consequently, no virological assays were performed on these samples.

## 3. Eosinophils in Tumor Biology: Recruitment, Prognostic Role, and Viral Context

### 3.1. Tumor-Associated Tissue Eosinophilia (TATE) and Tumor-Associated Blood Eosinophilia (TABE) in Solid Tumors

The terms TATE and TABE were introduced by Lowe et al. [17] to draw attention to the potential clinical and biological function of eosinophils in patients with neoplasms. TATE is characterized by eosinophil infiltration within tumor tissue, as determined by histological examination, whereas TABE refers to an increased eosinophil count in the peripheral blood, typically exceeding 3–5% of peripheral blood leukocytes, corresponding to an absolute eosinophil count of 0.35–0.5 × 10^9^/L, with slight laboratory-dependent variations [31]. TATE and TABE may occur either simultaneously or independently in different tumor types and anatomical locations [17]. Such findings support the concept that eosinophil recruitment is regulated by both local and systemic signaling pathways.

### 3.2. Local Mechanisms of Eosinophil Recruitment in Solid Tumors

Cormier et al. [32] used a mouse model of melanoma to study the mechanisms that regulate eosinophil infiltration in tumor sites. Histological analysis of these experimental tumors revealed that they had necrotic regions surrounded by living tumor tissue, with fibrous capsules separating the tumor from the surrounding host tissue. Eosinophil infiltration constituted an early event in tumor development, showing a preferential accumulation in both the fibrous capsule and necrotic areas. These findings suggest that zones undergoing necrosis and tissue remodeling primarily attract eosinophils. Immunohistochemical staining for major basic protein demonstrated diffuse extracellular matrix deposition in the necrotic areas, indicating that eosinophil degranulation was localized to these regions and absent from the capsule. Although eosinophil degranulation is primarily observed in necrotic tumor areas, this localization does not necessarily reflect a passive response to tissue damage. Instead, eosinophils might play an active role in harming tumor cells by releasing toxic proteins, which could help continue or increase the tissue damage. Experimental findings of Cormier et al. [32] served as a foundation for LIAR (Local Immunity and Remodeling) hypothesis, proposed by Lee et al. [33]. The LIAR hypothesis suggested that cell death, high mitotic activity, and possibly cancer stem cell activity in high-grade solid tumors represent potential foci for eosinophil recruitment. Accordingly, dying tumor cells release damage-associated molecular patterns, such as high-mobility group box 1 and IL-33, which promote eosinophilic infiltration. At the same time, proliferating tumor cells secrete factors that enhance eosinophil survival and differentiation, thereby sustaining local eosinophil presence [33]. Lee et al. [33] noted that TATE demonstrates significant variability, both between different types of tumors and within the same tumor throughout time. Their observations suggest that necrosis and proliferation, although potentially significant, are insufficient alone to account for eosinophil recruitment [19]. Additional factors, particularly tumor-derived eosinophil-specific chemotactic signals, must be considered. Among these, eotaxins, especially CCL11, play a crucial role in selectively attracting eosinophils to inflammatory and neoplastic sites by binding to CCR3 on eosinophils [34,35,36]. Cytokines such as IL-33 and IL-31 have recently been implicated in eosinophil recruitment and activation within the tumor microenvironment [37,38,39,40,41]. In summary, the mechanisms that regulate TATE are still not fully comprehended; the process is multifaceted and reliant on a variable combination of tumor hypoxia, cytokines, adhesion molecules, and chemokines in the tumor microenvironment. However, these microenvironmental mechanisms are merely one level of regulation, as systemic signals can also influence eosinophil recruitment and function.

### 3.3. Systemic Mechanisms of Eosinophil Recruitment and Regulation in Solid Tumors

Beyond local factors, systemic signals, particularly those mediated by endocrine hormones, may critically influence eosinophil biology in cancer. Estrogen signaling represents a paradigmatic example. In murine models of breast cancer, including triple-negative subtypes, and melanoma, Artham et al. [42] demonstrated that estrogen signaling decreased both TABE and TATE, thereby promoting tumor growth. In healthy female mice, estrogens reduced the number of peripheral eosinophils by limiting the proliferation and survival of maturing eosinophils. Conversely, pharmacological inhibition of estrogen receptors reduced tumor growth in an eosinophil-dependent manner and enhanced the efficacy of immune checkpoint blockade. These findings identify estrogen signaling as a systemic regulator of TATE and suggest that estrogen receptor modulators may serve as adjuvants to improve immunotherapy responses. Taken together, they reveal that systemic hormonal factors influence eosinophil recruitment, activation, and degranulation in solid tumors, in addition to local regulatory mechanisms.

### 3.4. Prognostic Significance of TATE in Human Malignancies

A growing body of evidence suggests that TATE is a favorable prognostic marker in several solid malignancies including gastric [43,44], colorectal [45,46,47,48], nasopharyngeal [49], oral cavity and lip [50], oral tongue [51], esophageal [52], and laryngeal carcinomas [53], as well as in melanoma [54,55], small cell esophageal carcinoma [56], and breast cancer [57]. A meta-analysis by Hu et al. [58] confirmed that TATE is a favorable prognostic marker in solid tumors. The study also demonstrated an intriguing negative correlation between TATE and critical indicators of tumor aggressiveness, including lymph node metastasis, advanced staging, and lymphovascular invasion [58].

On the other hand, several studies have shown that eosinophils may exert pro-tumorigenic effects in specific settings. In cervical squamous cell carcinoma, histologically assessed TATE has been associated with poor prognosis [59,60]. In contrast, studies on HPV-positive anal and oropharyngeal carcinomas evaluated TABE, not TATE [61]. In these settings, TABE were identified as independent negative prognostic factors for disease-free survival [61]. Interestingly, in HPV-negative oropharyngeal carcinomas, increased TABE levels were instead associated with improved prognosis [61]. In hematologic malignancies, TATE is present in 55–62% of classic Hodgkin lymphoma cases [62,63], whereas TABE occurs in about 15% [64]. In adult T-cell leukemia/lymphoma (ATLL), TABE was observed in approximately 9.5% of patients [65].

It is important to recognize that all these neoplasms share a viral genesis. HPV is implicated in the vast majority of anal (88%) [66], cervical (over 95%) [67,68], vaginal (60%) [69], vulvar (approximately 70%) [70], penile (over 50%) [71] squamous cell carcinomas. In HPV-associated tumors, viral oncoproteins skew the immune response toward a Th2 phenotype and stimulate the production of immunosuppressive and pro-angiogenic factors such as TGF-β, MMPs, and VEGF. Eosinophils, in this context, are recruited into the tumor stroma and may amplify these signals, thereby supporting tumor growth and neovascularization [72]. Approximately 40% of Hodgkin’s lymphoma cases are associated with Epstein–Barr virus (EBV) [73]. In EBV-positive Hodgkin lymphoma, latent viral proteins such as LMP1 activate signaling cascades including NF-κB and STAT3, leading to the release of eosinophil-attracting chemokines (e.g., CCL5, CCL28) and cytokines (e.g., IL-5, TGF-β) [74,75]. Eotaxin has been found to be expressed at a greater rate in Hodgkin lymphoma, and the amount of eotaxin in tissue has been shown to correlate with the extent of tissue eosinophilia. The result is a Th2-dominant microenvironment marked by immunosuppression, fibrosis, and inflammation that supports tumor persistence and expansion [74,75]. Similar mechanisms have been described in ATLL, a mature T cell neoplasm causally associated with human T-cell lymphotrophic virus type 1 (HTLV-1), where TABE is an independent unfavorable prognostic marker [65].

**Figure 2:**
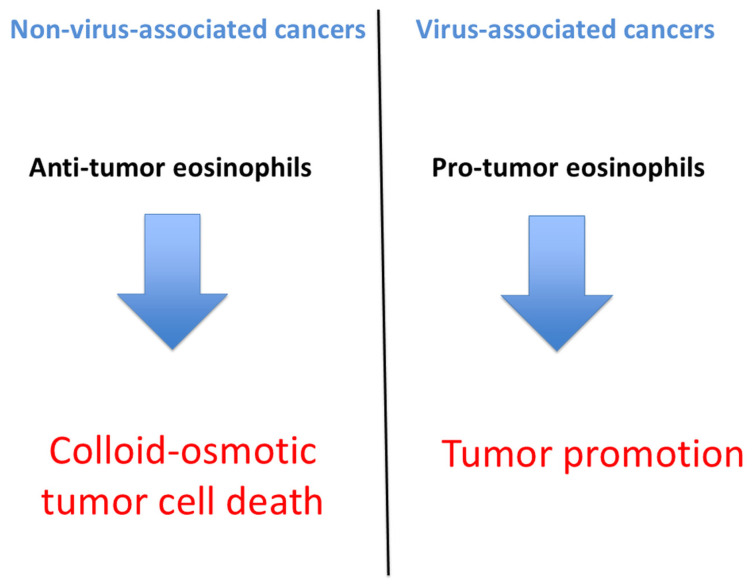
Schematic representation of the proposed dichotomy between virus-associated cancers and non-virus-associated cancers. In virus-associated tumors, eosinophils mainly exert pro-tumor functions, sustaining inflammation and contributing to tumor promotion. In contrast, in non-virus-associated tumors, eosinophils display anti-tumor activity, with polarized degranulation leading to colloid-osmotic tumor cell death.

The apparent contradiction in prognostic role of TATE and/or TABE likely reflects differences in the mechanisms of eosinophil recruitment within the tumor microenvironment. In virus-associated cancers, such as HPV-related carcinomas (cervical, anal and oropharyngeal squamous cell carcinomas), EBV-positive Hodgkin lymphoma, and HTLV-1 linked ATLL, eosinophilic infiltration is co-opted by the tumor to promote immune evasion, angiogenesis, and fibrosis, ultimately correlating with a poorer prognosis. Conversely, in tumors of non-viral etiology, eosinophil recruitment may be evoked by ischemic tumor injury, cell proliferation as well as by chemokines and cytokines and is associated with direct cytotoxic activity against tumor cells, contributing to a more favorable clinical outcome (Figure 2).

### 3.5. Dual Role of Eosinophils in Viral and Non-Viral Tumors

The role of eosinophils in virus-associated neoplasms is outlined in Table 1. Marked eosinophilic infiltration has been associated in some reports with reduced disease-free survival in classical Hodgkin lymphoma [62,63]. The cases of Hodgkin lymphoma associated with TATE have not been specifically investigated in relation to EBV infection. Although EBV positivity is a recognized prognostic factor in Hodgkin lymphoma [76], no studies have evaluated its interaction with TATE. Therefore, Hodgkin lymphoma was not included in Table 1, which summarizes virus-associated tumors where TATE/TABE has been analyzed in relation to prognosis. Future studies should include histological evaluation of eosinophilic infiltration and standardized viral testing to gain a better understanding of the prognostic impact of TATE in virus-driven neoplasms.

## 4. ETosis: A Distinct Mechanism of Eosinophil Degranulation

### 4.1. ETosis: Brief Overview

Eosinophils utilize four main degranulation pathways to release their granule contents: (1) Classical exocytosis, where individual granules fuse directly with the plasma membrane to discharge their mediators; (2) Compound exocytosis, involving prior granule–granule fusion followed by fusion with the plasma membrane to form large release channels; (3) Piecemeal degranulation, a selective and gradual process whereby granule-derived proteins are transported via eosinophil sombrero vesicles to the extracellular space, in the absence of granule fusion. In this process, the membranes of the specific granules remain intact, giving them the appearance of emptied containers; (4) Cytolysis, a lytic form of degranulation culminating in plasma membrane rupture and extracellular deposition of intact free eosinophil granules (FEGs) [3].

In 2004, Brinkmann et al. [77] discovered a new type of neutrophil cell death. This type of cell death is marked by the release of extracellular traps composed of decondensed chromatin and proteins from neutrophils, including histones, myeloperoxidase, and elastase [77]. This distinctive process, known as neutrophil ETosis, is mechanistically and morphologically distinct from apoptosis. Apoptosis is a tightly regulated, non-inflammatory form of programmed cell death marked by cytoplasmic shrinkage and well-defined nuclear changes such as chromatin condensation (pyknosis) and DNA fragmentation, whereas nuclear envelope remains structurally intact, although the nucleolus becomes unrecognizable [78,79,80]. In contrast, neutrophil ETosis involves early disintegration of the nuclear envelope and extensive chromatin decondensation that spills into the cytoplasm [77]. The plasma membrane then breaks and chromatin nets, full of antimicrobial components, are released in the extracellular space [77]. Unlike apoptosis, neutrophil ETosis does not cause nuclear fragmentation or the production of apoptotic bodies. Instead, it releases extremely pro-inflammatory DNA–protein complexes into the area outside the cells [77].

Although ETosis was originally described in neutrophils, subsequent studies have demonstrated that this form of cell death also occurs in other immune cells, including eosinophils, mast cells, plasmacytoid dendritic cells, basophils, monocytes, and macrophages [81]. In 2008, Yousefi et al. [82] reported that viable eosinophils can actively expel extracellular DNA traps of mitochondrial origin. In contrast to this report, Ueki et al. [11] demonstrated the discharge of similar DNA traps from cytolytic eosinophils. Expelled DNA was associated with histones, indicating a nuclear rather than mitochondrial origin [11]. As human eosinophils contain few mitochondria and mitochondrial DNA lacks histone organization, current evidence suggests that eosinophil ETosis is of nuclear rather than mitochondrial origin [83]. These findings have stimulated growing interest in the role of eosinophil ETosis in various non-neoplastic eosinophilic disorders [84].

### 4.2. ETosis in Non-Neoplastic Eosinophilic Disorders

Neves et al. [15] used TEM to examine tissue samples from patients with eosinophilic disorders such as eosinophilic chronic rhinosinusitis, ulcerative colitis, and hypereosinophilic syndrome. Their study revealed that eosinophil ETosis occurs at two different stages: an early phase and late phase [15]. In the initial stages of ETosis, nuclear lobes undergo chromatin decondensation, which eliminates the distinction between euchromatin and heterochromatin. Delobulation and rounding phenomena occur concurrently. In particular, nuclear lobes coalesce into a single rounded mass. As chromatin condenses and expands, the nuclear envelope progressively disintegrates, permitting its contents to pass into the cytoplasm. Concurrently, some cytoplasmic granules are extruded via a budding mechanism, still partially enveloped by remnants of the plasma membrane. In the advanced phase, rupture of the plasma membrane results in the abrupt release of decondensed chromatin into the extracellular space, where it forms extracellular traps, accompanied by the discharge of morphologically preserved FEGs, Charcot-Leyden crystals, and intact eosinophil sombrero vesicles [15,85].

Although eosinophil ETosis shares with neutrophil ETosis its pro-inflammatory nature and the formation of web-like chromatin structures, it differs markedly in granule dynamics: while neutrophils release enzymes such as elastase and myeloperoxidase due to granule disintegration [77], eosinophil traps are typically associated with intact granules [15,85]. The key morphological and functional differences among apoptosis, neutrophil ETosis, and eosinophil ETosis are summarized in Table 2.

### 4.3. Our Ultrastructural Observations of Eosinophil ETosis in Gastric Carcinoma

**Figure 3:**
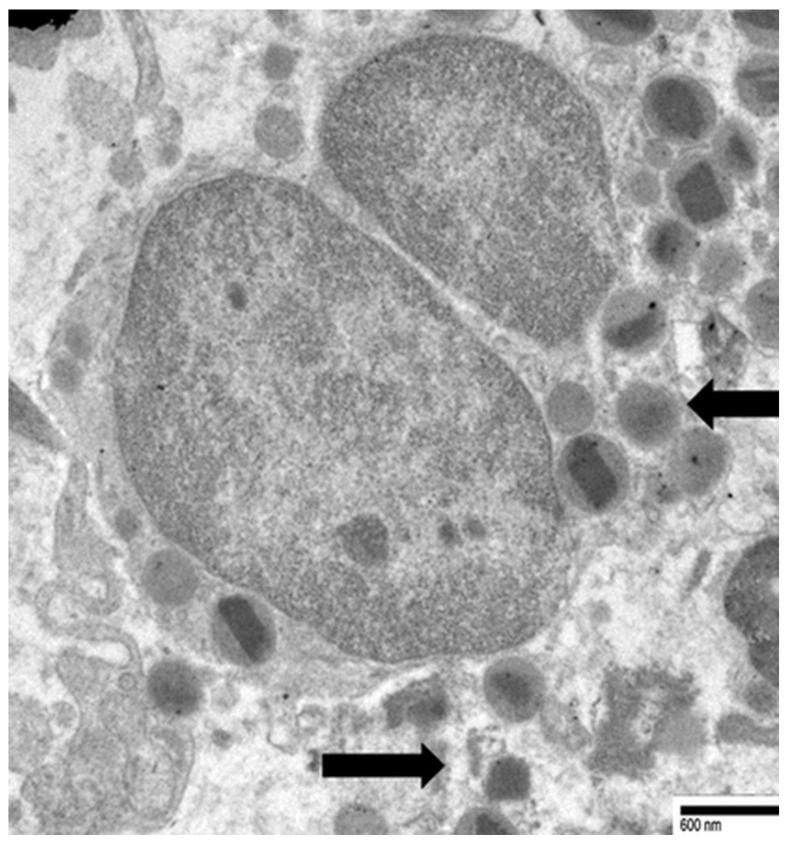
Electron micrograph of an eosinophil showing ultrastructural signs of cytolysis such as rounded nuclear lobes, chromatin decondensation and the release of FEGs (black arrows).

Neves et al. [15] showed that in non-neoplastic eosinophil-associated disorders, not all cytolytic eosinophils had characteristics of ETosis, suggesting that this process may be limited to a distinct subset of activated eosinophils. Our ultrastructural investigations in gastric malignancies with TATE [30] confirmed and extended the processes of eosinophil ETosis described in non-neoplastic eosinophil-associated disorders. In particular, in three cases of gastric cancers, we observed cytolytic eosinophils without ETosis. These eosinophils exhibited focal plasma membrane disruption, with release of FEGs, that retain their biphasic structure- dense core and surrounding matrix—and are scattered among collagen fibers near tumor cells. The nucleus maintained its bilobed morphology, with incomplete heterochromatin decondensation, and the nuclear envelope appeared mostly preserved or only mildly dilated (Figure 3).

**Figure 4:**
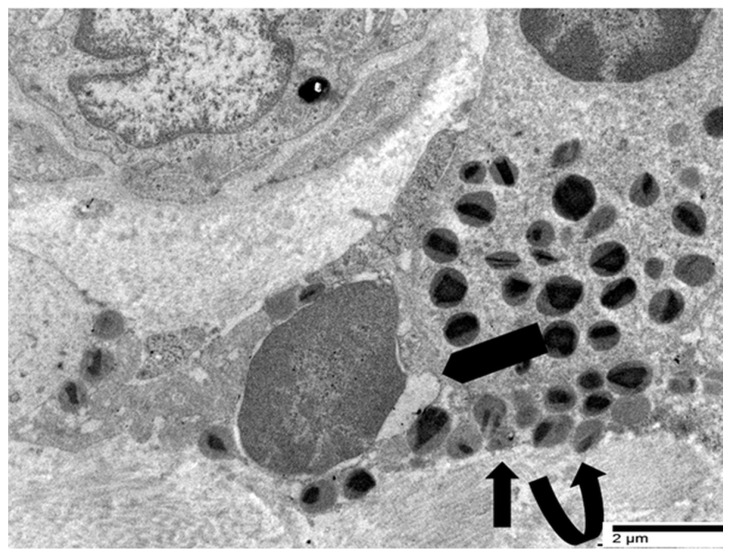
Electron micrograph of an eosinophil showing ultrastructural signs of early ETosis, including a slightly irregular profile of nuclear lobe, chromatin decondensation, dilation of nuclear envelope (pentagonal arrow), focal disruption of plasma membrane (black arrow), and the release of specific granules (curved arrow).

Cytoplasmic chromatin diffusion, Charcot-Leyden crystals, and extracellular trap formation were not observed [30]. In one case of gastric cancer, eosinophils exhibited ultrastructural features compatible with the distinct phases of ETosis, in line with the established dichotomy of intracellular and extracellular stages. Building on this paradigm, we suggested a more accurate three-stage ultrastructural classification that includes the spectrum of morphological changes that occur during ETosis: early, moderate, and advanced [30]. Figure 4 illustrates the early phase of ETosis, corresponding to the initial intracellular stage. The eosinophil displays rounded or oval nuclear lobes with considerable chromatin decondensation during the early stages of ETosis. The nuclear envelope is largely intact, although early signs of perinuclear space expansion are seen.

**Figure 5:**
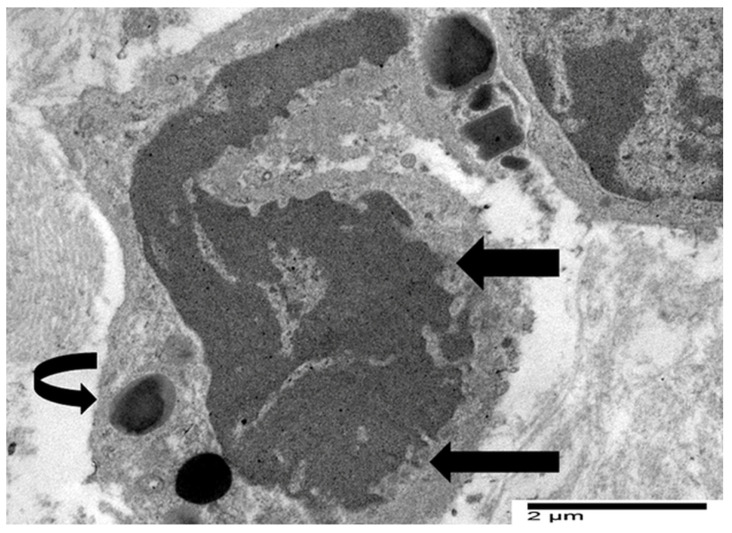
Electron micrograph depicting an intermediate phase of eosinophil ETosis. A few specific granules are present in the eosinophil (curved arrow), while decondensed chromatin appears as a cytoplasmic mass with irregular outlines (black arrows).

**Figure 6:**
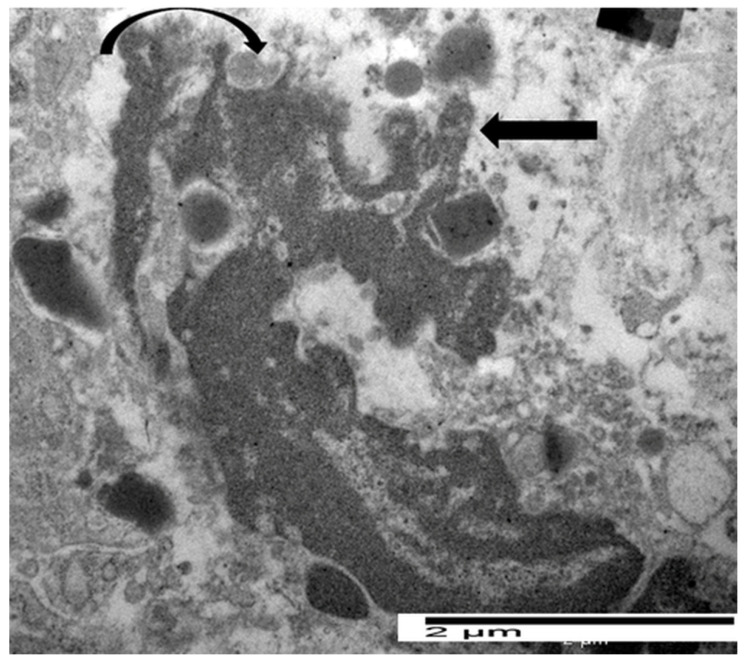
Electron micrograph illustrating an advanced stage of eosinophil ETosis. Extracellular DNA traps appear as decondensed chromatin structures with finger-like projections (curved arrow) or as sinuous, filamentous strands (black arrow), often located near FEGs.

Focal discontinuities in the plasma membrane are observed, together with the release of FEGs. Figure 5 shows the intermediate stage of ETosis, which is characterized by partial or whole nuclear envelope breakdown. An important ultrastructural characteristic of this stage is the change in nuclear morphology: whereas early-stage ETotic eosinophils retain rounded or oval nuclear lobes, the intermediate stage is characterized by a loss of nuclear symmetry, with lobes acquiring more elongated configurations and irregular, jagged contours, which reflect ongoing nuclear envelope breakdown and chromatin redistribution in the cytoplasm. The cytoplasm is either devoid of specific granules or it contains a limited quantity. Figure 6 illustrates the advanced stage of ETosis, which corresponds to the extracellular stage. This phase is characterized by complete rupture of the plasma membrane and massive release of decondensed chromatin into the extracellular space, forming extracellular traps. These chromatin networks often entangle FEGs and may extend toward or come into direct contact with neighboring tumor cells. Charcot-Leyden crystals may also be identified in proximity of ETotic eosinophils [30].

It is important to emphasize that eosinophil cytolysis, with or without ETosis, leads to the extracellular release of intact granules [15,30]. These granules, capable of ligand-induced secretion, operate as independent secretory organelles and can enhance the effector actions of eosinophils in the tumor microenvironment [86,87]. The extracellular deposition of FEGs may be regarded as a “minefield” with potential anticancer activity [30]. Although TATE is frequently focal and rarely characterized by dense infiltration, the cytotoxic potential of eosinophils may be markedly enhanced through the release of FEGs via cytolytic mechanisms [29].

## 5. Eosinophil-Mediated Tumor Cytotoxicity and Immune Synapse Formation

### 5.1. Eosinophil-Mediated Tumor Cytotoxicity: From In Vitro Mechanisms to In Vivo Evidence of Immune Synapse Formation

A landmark study by Legrand et al. [88] was the first to demonstrate that human eosinophils can kill colorectal cancer cells through contact-dependent processes and the release of soluble cytotoxic mediators. The authors used the Colo-205 cell line to show that eosinophils may induce rapid tumor cell apoptosis and to a lesser extent necrosis through cell–cell interactions mediated by the integrin complex CD11a/CD18. Eosinophils released a combination of effector molecules when they attached to tumor cells. The substances included eosinophil cationic protein, eosinophil-derived neurotoxin, TNF-α, and, importantly, granzyme A. The identification of granzyme A as part of the eosinophil cytotoxic repertoire is particularly important, as it mediates tumor cell death through a caspase-independent pathway and acts synergistically with eosinophil cationic protein and TNF-α [88]. Gatault et al. [89] subsequently revealed that IL-18 was a critical factor in the improvement of eosinophil–tumor cell interactions. IL-18 was produced by eosinophils when they came into contact with tumor cells and was essential for cytotoxic activity. Neutralization of IL-18 markedly reduced eosinophil-mediated Colo-205 apoptosis and inhibited eosinophil-tumor cell adhesion [89]. Following the in vitro demonstrations of eosinophil-mediated tumor cytotoxicity by Legrand et al. [88] and Gatault et al. [89], Andreone et al. [90] extended this line of research to in vivo models, offering crucial mechanistic insights into eosinophil function within the tumor microenvironment. In a melanoma-bearing mouse model, they showed that IL-33, an alarmin released by tumor cells in response to stressors such as hypoxia, promotes eosinophil recruitment, activation, and antitumor activity. IL-33-activated eosinophils established stable immune synapses with tumor cells and underwent polarized degranulation. The authors used TEM and confocal microscopy to demonstrate that granule convergence and focal release of cytotoxic mediator, such as eosinophil cationic protein, eosinophil peroxidase, and granzyme B, occurred at the eosinophil–tumor cell interface. The results provided direct morphological evidence of contact-dependent eosinophil cytotoxicity in vivo, culminating in tumor cell apoptosis [90].

### 5.2. Ultrastructural Evidence of Eosinophil-Mediated Tumor Cell Injury in Gastric Carcinomas

**Figure 7:**
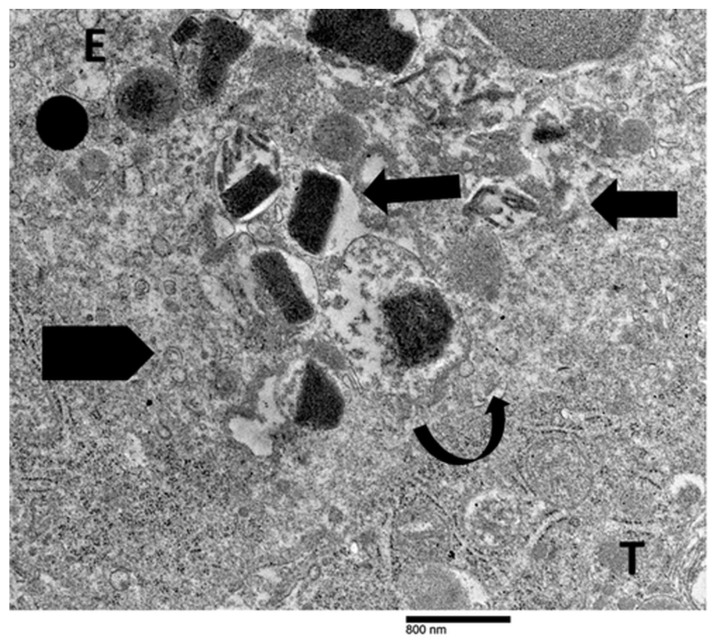
Ultrastructural image showing the interaction between tumor cell (T) and eosinophil (E). Fused specific granules (black arrows), indicative of compound exocytosis, are observed in the cytoplasm next to the tumor cell. Sparse sombrero vesicles are also observable (pentagonal arrow). The tumor cell and eosinophil exhibit discontinuities of plasma membrane (curved arrow).

In our previous ultrastructural investigations of gastric cancer specimens with elevated TATE, we focused on the interaction between eosinophils and tumor cells [28,29]. Eosinophils in close proximity to tumor cells in a single case of poorly differentiated tubular adenocarcinoma showed compound exocytosis (Figure 7) [28]. TEM revealed that fused specific granules and eosinophil sombrero vesicles were polarized toward the eosinophil–tumor cell interface, indicating a spatially targeted, regulated secretory response (Figure 7).

**Figure 8:**
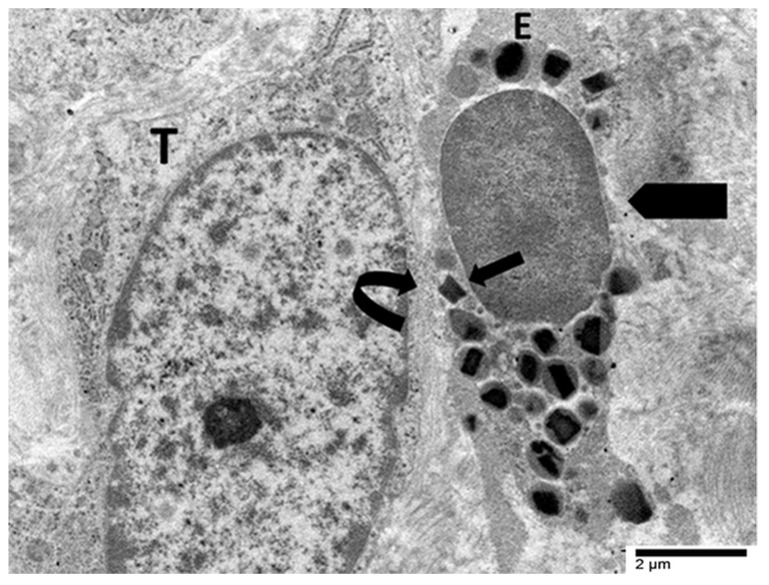
Electron micrograph showing the interaction between an eosinophil (E) and a tumor cell (T). The eosinophil displays initial ultrastructural signs of ETosis, including a rounded nuclear lobe, chromatin decondensation (pentagonal arrow), and the degranulation of specific granules near the tumor cell contact site (black arrow). The tumor cell exhibits a discontinuity in the plasma membrane at the site of eosinophil degranulation (curved arrow), suggesting a potential sublethal effect.

**Figure 9:**
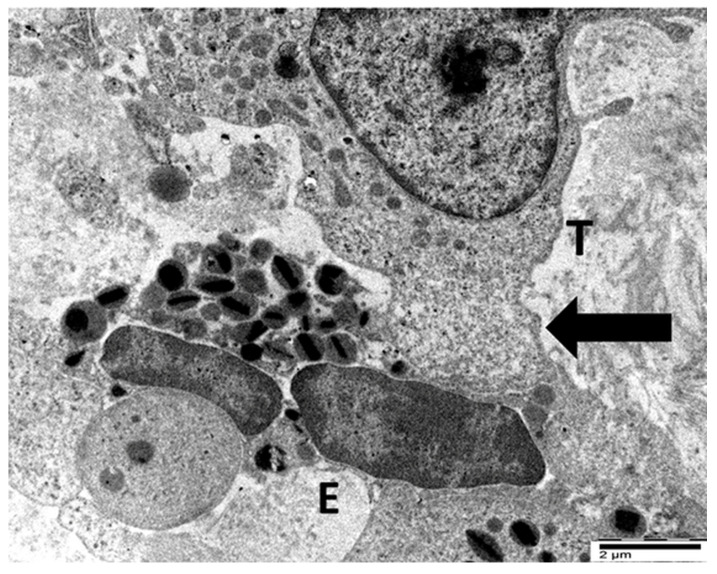
Electron micrograph illustrates an eosinophil (E) exhibiting early ETosis. The adjacent tumor cell (T) shows numerous cytoplasmic microvacuoles (arrow) in proximity to the contact area with ETotic eosinophil.

**Figure 10:**
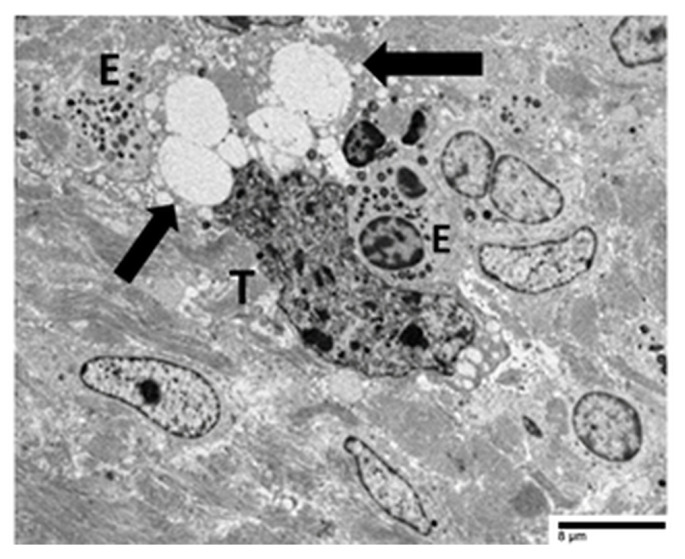
Heterotypic interaction between eosinophils (E) and a tumor cell (T). Numerous large cytoplasmic vacuoles (black arrows) are identified within the tumor cell cytoplasm adjacent to eosinophils. The presence of these vacuoles suggests colloid-osmotic tumor cell death, likely induced by eosinophils.

It is important to note that no evidence of eosinophil cytolysis was observed in this case. On the other hand, ETosis seemed to be the main eosinophilic degranulation mechanism in one case of poorly cohesive gastric carcinoma (not otherwise specified) (Figure 4, Figure 5 and Figure 6 and Figure 8, Figure 9 and Figure 10) [29].

These examples collectively illustrate two distinct modalities of eosinophil-mediated anticancer activity: (1) a contact-dependent, polarized exocytotic degranulation and (2) a contact-dependent, ETosis. Even though the patterns of eosinophil degranulation were different, similar ultrastructural features of tumor cell damage were noted in both cases. Importantly, tumor cells exhibited a range of ultrastructural changes that suggested progressive damage [28,29]. These changes could be various stages of eosinophil-induced cytotoxicity. Early damage was characterized by localized discontinuities in the tumor cell membrane at the site of contact with the eosinophils (Figure 7 and Figure 8). Figure 9 illustrates a scenario where cytoplasmic microvacuoles of the tumor cell occurred close to the eosinophil contact site. The most severe damage, as depicted in Figure 10, includes mitochondrial swelling, numerous large cytoplasmic vacuoles, and dilation of the nuclear envelope. These ultrastructural results support a model in which both eosinophil ETosis or exocytotic degranulation contribute to kill tumor cell through a non-apoptotic, colloid-osmotic mechanism (Figure 9 and Figure 10). The process progresses from eosinophil infiltration and direct cell-cell contact to membrane damage and cytoplasmic vacuolation in tumor cells [28,29]. Although molecular assays were not performed on these archival samples, our ultrastructural investigations did not reveal any of the characteristic features of viral infection, such as intranuclear viral capsids, cytoplasmic budding virions, or paracrystalline arrays [91,92]. The absence of these ultrastructural findings suggests that the examined gastric carcinomas were not associated with viral infection, thereby supporting the main hypothesis of this review that TATE generally conveys a favorable prognostic significance in non-viral cancers.

Several important characteristics of the murine melanoma model described by Andreone et al. [90] are shared by our observations, such as the polarization of eosinophilic granules and the formation of synapse-like junctions between eosinophils and tumor cells. Nevertheless, the nature of tumor cell death is a critical distinction: our results indicate that necrosis is the predominant consequence, in contrast to the apoptosis induced by eosinophils reported by Andreone et al. [90]. To better contextualize our ultrastructural observations within the framework of experimental studies, we provide a comparative overview in Table 3, which highlights the mechanistic similarities (eosinophil–tumor synapse formation, polarized degranulation) and the differences in tumor cell death pathways (apoptosis versus colloid-osmotic necrosis).

In our previous ultrastructural studies on gastric carcinomas without TATE, such as parietal cell-differentiated [93], hepatoid [94], mitochondria-rich [95], and pleomorphic giant cell variants [96], as well as tumors showing granulomatous [97], or autophagic features [98], eosinophil-tumor synapses and colloid-osmotic necrosis were not observed, indicating that these features are restricted to eosinophil-rich gastric cancers.

**Figure 11:**
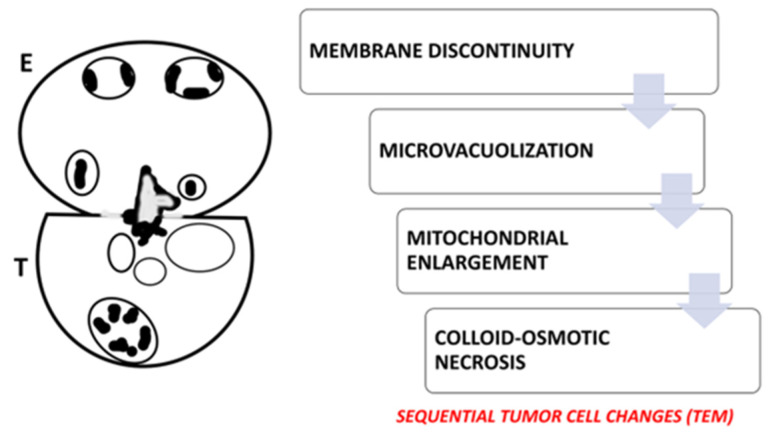
Schematic representation of the eosinophil (E)-tumor cell (T) synapse-like interaction. The eosinophil in contact with the tumor cell exhibits polarized granule convergence and compound exocytosis at the eosinophil-tumor cell interface. The tumor cell shows features of colloid-osmotic cell death, including mitochondrial enlargement and cytoplasmic vacuolation. The adjacent flow chart illustrates the sequential changes in the tumor cell: membrane discontinuity, microvacuolization, mitochondrial enlargement, and colloid-osmotic cell death.

Our ultrastructural findings, which include polarization of specific granules, synapse-like contacts, and localized membrane disruption, collectively provide strong support for a model of contact-dependent eosinophil-mediated tumor cell necrosis in vivo in gastric carcinomas (Figure 11). These ultrastructural data emphasize the potential relevance of eosinophils as effector cells in the tumor microenvironment and necessitate further investigation into their role in antitumor immunity.

## 6. Limitations

This review was performed as a narrative synthesis of the literature rather than a systematic review with meta-analysis. The quantitative significance of the reported associations between TATE and prognosis should be interpreted with caution, as no pooled statistical estimates were calculated. There is also the possibility of publication bias, as studies that report positive or confirmatory results are more likely to be published than those that present null or unclear data. This may alter the dominant perception of TATE as a generally favorable prognostic marker. However, the integration of original ultrastructural data from gastric carcinoma cases constitutes a distinctive strength of our review. The gold standard to describe eosinophil-specific granules and degranulation patterns is still TEM because it provides a high-resolution view of ETosis and eosinophil degranulation, thereby offering important morphological insights. Future investigations that integrate ultrastructural, immunophenotypic, and functional assessments in larger cohorts are essential for confirming the prognostic role of eosinophils in the tumor microenvironment.

## 7. Conclusions and Future Directions

Eosinophils have been mainly studied through a dichotomous lens, either as pro-tumorigenic or anti-tumorigenic effectors. This has led to in the prevailing view that their role in cancer is inherently contradictory. However, based on our recent findings, we propose an alternative interpretive paradigm in which the functional orientation of eosinophils in tumor immunology may be strongly influenced by the presence or absence of oncogenic viral infections, particularly HPV. TATE and/or TABE have been associated with poorer clinical outcomes in HPV-related malignancies, including cervical, anal, and oropharyngeal squamous cell carcinomas. HPV may drive eosinophils toward a phenotype that supports tumor growth, thereby contributing to disease progression. The predictive significance of TATE in Hodgkin lymphomas remains debated, and further studies are required to clarify the potential impact of EBV infection on its prognostic value. Although several reports suggest that TATE may be associated with a favorable prognosis in non-virus-related cancers, the findings are heterogeneous and sometimes conflicting, and the lack of meta-analytic evidence prevents definitive conclusions. These observations highlight the need to consider the viral context in future investigations aimed at elucidating the immunological functions of eosinophils in the tumor microenvironment.

Our ultrastructural analysis revealed close contact between eosinophils and tumor cells in gastric cancers exhibiting TATE. These contacts were associated with focal disruption of the tumor cell plasma membrane, followed by a series of cytopathic changes, including mitochondrial swelling, cytoplasmic vacuolization, and nuclear envelope dilation, indicative of a non-apoptotic colloid-osmotic tumor cell death. The interaction between eosinophils and tumor cells is analogous to synapse-polarized eosinophil degranulation as described by Andreone et al. in their experimental studies [90]. Nevertheless, this interpretation is exclusively based on morphological evidence. Additional research employing immunoelectron microscopy or molecular markers of synaptic architecture is required to ascertain if the interactions between eosinophils and tumor cells represent true functional immune synapses.

Eosinophil ETosis has been identified as a mechanism contributing to tumor control in gastric carcinomas through the release of DNA traps and cytotoxic granule contents. The presence of eosinophil ETosis in tumor tissue offers valuable morphological information about the immune microenvironment and suggests a possible role in modulating tumor–immune interactions. Nevertheless, intratumoral eosinophil ETosis has yet to be systematically quantified, highlighting the need for further studies to better characterize this phenomenon.

## Figures and Tables

**Table 1:** Main virus-associated tumors, eosinophilia (TATE/TABE), and clinical outcomes.

Neoplasm	Virus Identification	Type of Eosinophilia	Clinical Outcome	Reference
Cervical squamous cell carcinoma	HPV	TATE	TATE increases with cancer progression	Xie et al. [60]
Cervical squamous cell carcinoma	Not performed	TATE	TATE predicts worse overall survival	Van Driel et al. [59] †
Anal squamous cell carcinoma	HPV	TABE	TABE is a negative independent prognostic factor for disease-free survival	Rimini et al. [61]
Oropharyngeal cancer	HPV	TABE	TABE is associated with worse disease-free survival	Rimini et al. [61]
Adult T-cell leukemia/lymphoma	HTLV-1	TABE	TABE is an independent unfavorable prognostic factor	Utsunomiya et al. [65]

† The study of Van Driel et al. [59] was included despite no HPV status verification, given the rarity (<5%) of HPV-negative cervical squamous cell carcinomas [67,68].

**Table 2:** Granulocyte Cell Death Modalities: Distinguishing Apoptosis, Neutrophil ETosis, and Eosinophil ETosis.

Feature	Apoptosis	Neutrophil ETosis	Eosinophil ETosis
Type of cell death	Programmed, non-inflammatory	Programmed, pro-inflammatory	Programmed, pro-inflammatory
Nuclear organization	Condensation and fragmentation	Delobulation and decondensation	Delobulation and decondensation
Nuclear envelope integrity	Preserved until late stages	Disrupted	Disrupted
Plasma membrane rupture	No	Yes	Yes
Cytoplasmic granules	Intact, not involved	Disintegrated; enzymes released into nucleus	Intact; associated with extracellular traps
Content of extracellular traps	Absent	DNA + elastase, myeloperoxidase, other granule-derived enzymes	DNA + morphologically intact specific granules

**Table 3:** Comparison between experimental studies and ultrastructural human observations of eosinophil-tumor cell interactions.

Model	Eosinophil-Tumor Cell Interaction	Eosinophil Cytoxicity	Tumor Cell Outcome	References
In vitro assays (colo-205 carcinoma cell line	Mediated by CD11a/C and IL-18	Degranulation and mediator release (eosinophil cationic protein, eosinophil-derived neurotoxin, TNF-alpha, granzyme A	Induction of apoptosis	Legrand et al. [88]; Gatault et al. [89]
In vivo murine melanoma	Mediated by IL-33	Synapse-polarized degranulation and mediator release (eosinophil cationic protein, eosinophil peroxidase, granzyme B	Induction of apoptosis	Andreone et al. [90]
Human gastric cancer TEM	Synapse-like interaction	Compound exocytosis or ETosis	Induction of colloid-osmotic death	Caruso et al. [28]; Caruso et al. [29]

## Data Availability

Not applicable.

## Abbreviations

The following abbreviations are used in this manuscript:

TATE	tumor-associated tissue eosinophilia
HPV	Human papillomavirus
HTLV-1	Human T-lymphotropic virus type 1
ETosis	Extracellular trap cell death
TEM	Transmission electron microscopy
TABE	Tumor associated blood eosinophilia
LIAR	Local Immunity and Remodeling
ATLL	Adult T-cell leukemia/lymphoma
EBV	Epstein–Barr virus
FEGs	Free eosinophil granules
